# An Evaluation of Occupational Stress and Periodontal Status Among Indian Army Male Personnel: A Cross-Sectional Study

**DOI:** 10.7759/cureus.62411

**Published:** 2024-06-14

**Authors:** Ajay Reddy Palle, Rinky Tripathi, Anahita Punj, Sanjay Vasudevan, Atchuta Abhinav, Manisha Bommala

**Affiliations:** 1 Periodontology, Army College of Dental Sciences, Secunderabad, IND

**Keywords:** risk factor, oral hygiene, gingivitis, surveys and questionnaires periodontitis, psychological, stress, occupational stress

## Abstract

Background: Oral illnesses are multifactorial and also behavior-related. Stress and habits influence periodontal disease. Based on this premise, assessing the influence of stress on military personnel and understanding its influence on oral and periodontal health becomes imperative.

Aim: The aim of this study was to evaluate the occupational stress and periodontal status among Indian army personnel.

Methods: A cross-sectional study was carried out among 1000 army personnel divided into four groups of 250 each. Group A had recruits, Group B had trainee officers, Group C personnel were serving in the army below the officer’s rank and Group D had officers who were currently serving in the army. Oral Hygiene Index - Simplified (OHI-S), Gingival Bleeding Index (GBI), pocket depth, and clinical loss of attachment (CLOA) were assessed for periodontal status. Stress levels were evaluated using the Modified Occupational Stress Index Questionnaire. Analysis of variance (ANOVA) test was applied to compare OHI-S, GBI, presence of pocket depth, and occupational stress scores, while the Kruskal-Wallis test was used to compare the occupational stress index and CLOA among the study groups.

Results: Significant differences were observed among the four groups in terms of OHI-S (p = 0.001), GBI (p = 0.001), CLOA (p = 0.001), occupational stress scores (p = 0.001), and occupational stress index (p = 0.001). Differences observed among the four groups regarding periodontal disease based on the presence of pockets were weakly significant (p = 0.05). On intergroup comparison, a significant association could not be established between the groups as the p-value was ≥ 0.05. However, a weak association could be established between Groups A and C with a p-value of 0.031. Participants with moderate to high occupational stress showed more risk of developing pockets and CLOAs as compared to those having low stress.

Conclusion: The study revealed that the majority of army personnel were suffering from gingival bleeding and periodontal diseases; however, the association between stress level and periodontal diseases in Indian army personnel was less and not significant.

## Introduction

The accumulation of bacteria on the teeth and their subsequent interaction with the human immune system leads to periodontal disease. Periodontitis is multifactorial and one such component is stress, which controls the body's immune response through the hypothalamic-pituitary-adrenal (HPA) axis and may be a contributor to gum disease [1]. Stress can have both direct and indirect effects on periodontium, both physiologically and psychologically. The direct effect involves modulation of the host defense system, producing immunosuppression and increasing susceptibility to illness [2,3]. Indirectly, detrimental lifestyle choices, such as not brushing your teeth, smoking, drinking too much alcohol, and eating poorly, may affect one’s mental health [4,5]. Notwithstanding these findings, it is still unclear how stress contributes to an increased risk of periodontal diseases.

Army soldiers are at a higher risk than the general population for developing oral problems owing to the unique psychological, physiological, and environmental stresses to which they are constantly subjected in the course of their duties [6,7]. The absence of normal living and working conditions and complex working patterns among army personnel often leads to irregular dietary practices, sleep disturbances, and the development of stress. Although they are trained to deal with such conditions, they are not immune to work stress-induced behavioral changes, and since oral diseases are behavior-related, some changes in the oral cavity could be anticipated. Very few studies in India have assessed the oral health status of army personnel in India [6,7]. A study previously on army personnel had divided the sample size into three groups based on their units. This study showed that the majority of army personnel had calculus deposits in all groups, followed by shallow pockets, bleeding on probing, and deep pockets [8]. Another study proved that many recruits have calculus followed by shallow pockets. Less than 10% had bleeding on probing and deep pockets [9]. There appears to be a physical and chemical connection between stress and its role in progression of periodontal disease in the presence of etiological factors. Stress can potentially change the biochemistry of the local periodontal microenvironment and global inflammatory burden in the body. Cortisolemia and increased inflammatory burden associated with stress have been seen correlated with periodontal disease [10]. Stress and its likely link to periodontal disorders among Indian military personnel have not been studied yet. The purpose of the current research was to assess occupational stress and periodontal conditions among Indian army troops across different hierarchical levels and age groups and to determine whether a relationship exists between the two.

## Materials and methods

Study population

One thousand men in the Indian Army between the ages of 18 and 50 were surveyed in a cross-sectional research conducted in Secunderabad, India. The sample size was calculated using the formula n = Z^2^p(1-p)/ ε^2^, where Z = Z score, ε is the margin of error set at 5%, n = population size, and p is the population proportion. Women were excluded as they were not equally distributed in the four groups to avoid bias. The research was conducted after receiving clearance from the institution's ethics board. The Indian Army granted centralized approval for this research to be conducted on its soldiers. Informed consent in accordance with the Declaration of Helsinki was obtained from the participants.

Inclusion and exclusion criteria

Systemically healthy participants aged 18-50 years having Angle’s Class 1 molar relationship were included as the prevalence of periodontal disease increases with age and stressors appear early in the studied population [11]. Participants with dental malocclusion - Angle’s Class 2 and Class 3 molar relationship and increased overjet/overbite - were excluded to avoid malocclusion as a confounding factor. Malocclusion is considered a risk factor for periodontal disease as it increases plaque accumulation [12]. Participants who had undergone periodontal treatment six months before the examination were also excluded. The participants were divided into four groups: Group A included recruits, Group B had trainee officers, Group C personnel were serving in the army below the officer’s rank, and Group D had officers who were currently serving in the army. The age distribution in the group was as follows: Groups A and B consist of individuals aged 18 to 22 years, whereas Groups C and D comprise individuals aged 23 to 50 years.

Armamentarium

A set of pre-sterilized 100 mouth mirrors and 100 specially designed, lightweight Community Periodontal Index (CPI) metallic probes with a 0.5-mm ball tip was used, with a black band between 3.5 and 5.5 mm, and rings at 8.5 and 11.5 mm from the ball tip. The ball-ended tip of 0.5 mm prevents excessive pressure while probing and maintains the force between 15 and 25 grams. The instruments, after use, were washed thoroughly with a scrubbing brush, soap, and water. Then the instruments were kept in a holding solution of glutaraldehyde (Korsolex Rapid® disinfectant solution; B. Braun, Melsungen, Germany) for 15 minutes to achieve cold sterilization. The solution was made by diluting five parts of Korsolex Rapid® solution into 95 parts of drinking water to make a 5% solution, as mentioned in the instruction manual of Korsolex Rapid. Instruments were then dried, kept in the portable autoclave (Life Dental & Clinical Autoclave ST911EPT With Drum - 11 Litres; Life Steriware, New Delhi, India), and subjected to steam sterilization for a cycle of 30 minutes to achieve complete sterilization.

Screening

Each participant was evaluated for periodontal condition for the following parameters: Oral Hygiene Index - Simplified (OHI-S) [13], Gingival Bleeding Index (GBI) [14], Periodontal Pockets, and Clinical Loss of Attachment (CLOA) [15]. Six sites on every tooth were examined and both the presence and depth of pocket were recorded. Pockets were scored as 0 = absence of condition, 1 = 4-5 mm pocket depth, 2 = 6 mm or more pocket depth. Six index teeth (16, 11, 26, 36, 31, 46) as per the Community Periodontal Index of Treatment Needs (CPITN) index were used for calculating CLOA. Two or more interproximal sites with a clinical attachment loss (CAL) of "greater than or equal to 6 mm" are required to fulfill the case criteria for severe periodontitis. These features need not all be located on the same tooth. CAL of "greater than or equal" to 4 mm at two or more interproximal sites (not on the same tooth) and probing depth (PD) of "greater than or equal" to 5 mm at two or more interproximal sites were considered indicative of moderate periodontitis [16]. A single investigator, well-versed in the factors under study, conducted all the exams. Ten additional military people who were not involved in the main research were used for calibration by administering identical tests to them. The intra-class correlation coefficient (ICC) found intra-examiner reliability for many measures to be between 0.83 and 0.91.

The stress levels of study participants were assessed using the Occupational Stress Index (OSI - 46 Item Questionnaire) created by Srivastava and Singh (1984) [17,18]. Ten new questions were developed and added for the Indian Armed Services after consultation with specialists from military backgrounds. Ten members of the military were given this revised version (now 56 items) on two separate occasions to evaluate test-retest consistency (see Appendices). The dependability index, or kappa, came in at 0.78. Using a modified version of the back-to-back translation method, the original OSI-56 English version was converted into a Hindi version. Disagreements were eliminated after checking the Hindi back-translations against the original English instruments. In the end, there were 56 questions total, 38 of which were "True keyed" and 18 false. Job overload, job ambiguity, job difficulty, group and political conflicts, the duty of individuals, under-interest, weakness, terrible companion relationships, inborn poverty, poor status, difficult working conditions, and unrewarding quality were all topics addressed in the survey. The scale is meant to quantify how much workers are stressed by different aspects and circumstances of their jobs. Each person's OSI score was calculated by adding the raw values of their replies to all 56 questions. The scores given for OSI were 5 for strongly agree, 4 for agree, 3 for undecided, 2 for disagree, and 1 for strongly disagree. To estimate the level of workers' occupational stress, we add up the scores on all the statements. Low occupational stress was defined as a score of 115 or less, moderate stress as 116-161, and severe stress as a score of 161 or more. OSI was used by Ugwuanyi et al. [19], in their research titled "Impacts of Cognitive Behavior Therapy on Occupational Stress Among Science and Social Science Education Facilitators in Open and Distance Learning Centers and Its Implications for Community Development: A Randomized Trial Group."

The data was compiled and analyzed using Statistical Package for the Social Sciences (IBM SPSS Statistics for Windows, IBM Corp., Version 20.0, Armonk, NY) by keeping the level of significance at 5%. Based on the distribution of data, parametric or non-parametric tests were applied accordingly. One-way analysis of variance (ANOVA) test was applied to compare OHI-S, GBI, presence of pocket depth, and occupational stress scores, while the Kruskal-Wallis test was used to compare the OSI and CLOA among the study groups as the values were not normally distributed. The post hoc Tukey's test was used for pairwise comparison of OHI-S, GBI, presence of pocket depth, and occupational stress scores. Mann-Whitney Test and post hoc Bonferroni test were used for pairwise comparison of CLOA and OSI respectively. The association of occupation stress at work with periodontal disease was assessed with a chi-square test. Binary logistic regression analysis was carried out to see how the two parameters for periodontal disease, pocket depth, and CLOA were related to occupational stress.

## Results

The results in Table 1 depict the demographic details of participants. The mean age of the study participants was 28.40 years with a standard deviation of 8.21, the mean number of teeth present was 31.11, and the majority of the participants (749 and 760) were not married and not staying with their family respectively.

**Table 1:** Demographic details of study participants N: number of participants; SD: standard deviation

Groups	N	Mean age ± SD	Marital status		Staying with family		Number of teeth present
			Married	Not married	Yes	No	
A	250	21.88 ± 1.05	146	104	52	198	31.10
B	250	20.52 ± 1.04	0	250	0	250	30.36
C	250	37.11 ± 4.72	5	245	105	145	31.83
D	250	29.67 ± 9.57	100	150	83	167	31.17
Total	1000	28.40 ± 8.21	251	749	240	760	31.115

Table 2 shows a comparison of clinical parameters among the study participants. ANOVA was used for comparing OHI-S, GBI, presence of pockets, and occupational stress scores. The difference in OHI-S score among the subject groups was significant (p = 0.001) with Group C having the highest OHI-S score (2.11 ± 0.79) and Group B showing the least OHI-S score (1.38 ± 0.64). Even pairwise comparison showed significant differences except when comparing Groups A and D. The difference in GBI score among the subject groups was significant (p = 0.001) with Group C having the highest GBI score (1.00 ± 0.28) followed by Group D (0.87 ± 0.21). Groups A and B showed the same GBI score (mean GBI = 0.86 each). Pairwise comparison showed significant differences were present between the pairs, A and C, B and C, and C and D. The number of teeth sites having pockets was observed more in Group C with pocket depth >3 mm (1.69 ± 3.47) followed by Group D participants (1.32 ± 3.39) and Group B participants (1.26 ± 2.33). Participants in Group A had fewer periodontal pockets (0.96 ± 2.49) and this difference in periodontal pockets among study participants was statistically non-significant (p = 0.220), but pairwise comparison showed significant differences between Groups A and C. Occupational stress mean scores were highest in Group A (175.34 ± 23.21), followed by Groups C (169.77 ± 21.70), B (161.70 ± 24.64), and D (155.15 ± 24.07). Pairwise comparison also revealed that the differences observed among the groups were significant (p < 0.05). The OSI also followed a similar pattern with Group A having the highest mean rank score followed by Groups C, B, and D and the differences observed were significant (p < 0.05). The Kruskal-Wallis test was used for comparing CLOA and occupational stress index. Significant differences (p = 0.001*) in CLOA were observed in all groups and the mean rank value was highest in Groups C and D participants. Mild loss of attachment loss was seen in Groups A and B participants. Further, pairwise comparison using the post hoc Bonferroni test for CLOA revealed significant differences between Groups A and B, B and C, and C and D except for tooth number 11. Significant pairwise differences were also observed between Groups A and D for tooth numbers 16, 26, 46, and 31.

**Table 2:** Comparison of clinical parameters (OHI-S, GBI, pockets, occupational stress scores, occupational stress index, and CLOA) among the four groups N: number of participants; NA: not applicable; SD: standard deviation; * indicates p<0.05 (significant); NS: not significant; # tooth number; OHI-S: Oral Hygiene Index-Simplified; GBI: Gingival Bleeding Index; CLOA: Clinical Loss of Attachment; ANOVA: analysis of variance

Clinical parameters	Group	N	Tooth no.	Mean ± SD	Mean rank	One-way ANOVA test	Kruskal-Wallis test	Pairwise comparison (post hoc Tukey's test/ Mann-Whitney U test/post hoc Bonferroni test) p-value					
						p-value		A vs B	A vs C	A vs D	B vs C	B vs D	C vs D
OHI-S	A	250	16, 11, 26, 31, 36, 46	1.73 ± 0.71	NA	0.001*	NA	0.001*	0.001*	0.334 (NS)	0.001*	0.001*	0.001*
	B	250		1.38 ± 0.64									
	C	250		2.11 ± 0.79									
	D	250		1.62 ± 0.70									
GBI	A	250	All teeth present	0.86 ± 0.26	NA	0.001*	NA	1.000 (NS)	0.001*	1.000 (NS)	0.001*	0.999 (NS)	0.001*
	B	250		0.86 ± 0.29									
	C	250		1.00 ± 0.28									
	D	250		0.87 ± 0.21									
Presence of Pockets	A	250	All teeth present	0.96 ± 2.49	NA	0.05	NA	0.670 (NS)	0.031*	0.536 (NS)	0.371 (NS)	0.997 (NS)	0.498 (NS)
	B	250		1.26 ± 2.33									
	C	250		1.69 ± 3.47									
	D	250		1.32 ± 3.39									
Occupational Stress Scores	A	250	NA	175.34 ± 23.21	NA	0.001*	NA	0.001*	0.040*	0.001*	0.001*	0.010*	0.001*
	B	250		161.70 ± 24.64									
	C	250		169.77 ± 21.70									
	D	250		155.15 ± 24.07									
Occupational Stress Index	A	250	NA	NA	586.83	NA	0.001*	0.001*	0.008*	0.001*	0.001*	0.001*	0.001*
	B	250			461.66								
	C	250			533.64								
	D	250			416.88								
CLOA	A	250	16	NA	478.39	NA	0.001*	<0.001*, 0.001* (#11)	1.000 (NS)	0.010* (#16), 1.000 (NS(#11)), 0.044* (#26), 0.010* (#46), 0.001* (#31), 0.337 (NS(#36))	<0.001*	0.622 (NS(#16)), 0.231 (NS(#11)), 1.000 (NS(#26)), 1.000 (NS(#46)), 1.000 (NS(#31)), 0.243 (NS(#36))	<0.001*(#16), 0.642 (NS(#11)), 0.001* (# 26,46), <0.001* (#31), 0.016* (#36)
			11		487.43								
			26		484.89								
			46		484.46								
			31		471.76								
			36		487.90								
	B	250	16	NA	460.48								
			11		485.44								
			26		464.99								
			46		472.49								
			31		471.76								
			36		468.00								
	C	250	16	NA	550.91								
			11		527.77								
			26		541.36								
			46		530.68								
			31		529.16								
			36		542.28								
	D	250	16	NA	512.22								
			11		501.36								
			26		510.76								
			46		514.37								
			31		529.32								
			36		503.82								

Table 3 evaluates the association of occupational stress with periodontal diseases. As compared to participants having low occupational stress, the odds of developing periodontal pockets were higher among the participants with moderate stress (OR: 2.578; 95% CI: 0.742-8.955) and participants with high stress (OR: 2.528; 95% CI: 0.732-8.736). The risk of CLOA was higher among the participants with moderate stress (OR: 1.583; 95% CI: 0.454-5.525) and participants with high stress (OR: 1.395; 95% CI: 0.402-4.843) as compared to participants having low occupational stress based on the odd's ratio mentioned above.

**Table 3:** Association of occupational stress with periodontal disease The analysis was conducted using binary logistic regression; Low stress was considered as the reference category; Pockets: Present/Absent; CLOA: Present/Absent

Dependent variable	Stress	Exp (β)	95% confidence interval	
			Lower bound	Upper bound
Pockets	Low (Ref)	-	-	-
	Moderate	2.578	0.742	8.955
	High	2.528	0.732	8.736
Clinical loss of attachment	Low (Ref)	-	-	-
	Moderate	1.583	0.454	5.525
	High	1.395	0.402	4.843

**Figure 1:**
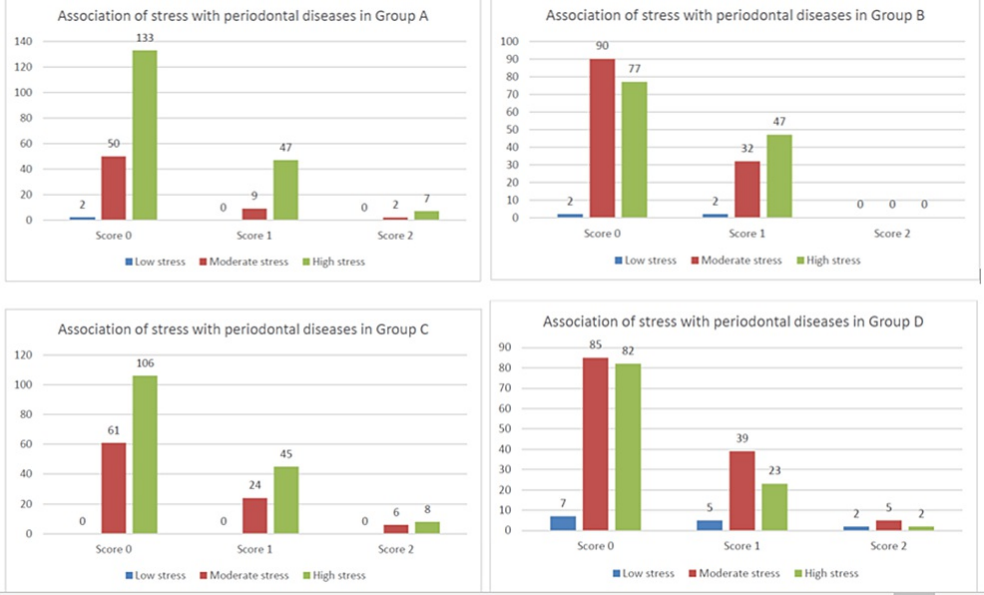
Association of occupational stress with periodontal disease Graphical representation of the association between occupational stress and periodontal disease based on periodontal disease severity score and occupational stress index.

Overall, there was a weak non-significant association between periodontal disease and occupational stress in each group which can be visualized graphically as depicted in Figure 1. It can be reiterated that high and moderate stress scores were associated with the severity of periodontal disease but the association is weak and non-significant in the population under study.

**Figure 2:**
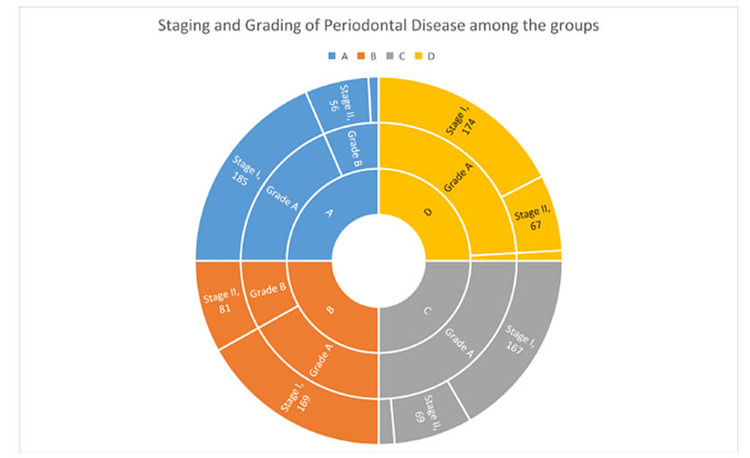
Staging and grading of periodontal disease among the four groups (A, B, C, and D) A, B, C, and D in the center represent the four groups and each group is classified retrospectively as per staging and grading of periodontal disease. Grade A: slow rate of bone loss i.e. <0.25 (% bone loss/age); Grade B: moderate rate of bone loss i.e. 0.25-1 (% bone loss/age); Stage I: interdental CAL = 1-2 mm, probing depth ≤4 mm; Stage II = interdental CAL = 3-4 mm, probing depth ≤5 mm; and Stage III = interdental CAL ≥ 5 mm, probing depth ≥ 6 mm. CAL: clinical attachment loss

The periodontal disease in the studied population on retrospective analysis revealed having a slow to moderate bone loss pattern and staging of the disease ranging from I to III according to the 2017 classification [20] as shown in Figure 2.

**Figure 3:**
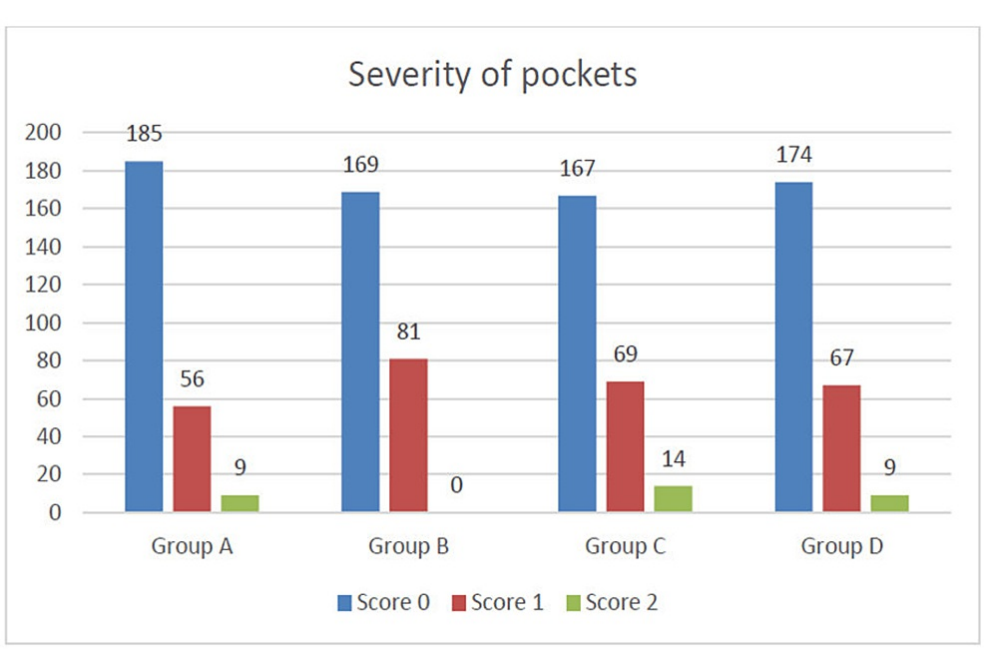
Assessment of severity of periodontal disease based on pocket depth among all the groups Score 0 = Absence of condition, Score 1 = 4-5 mm pocket, Score 2 = 6 mm or more pocket

Moreover, Figure 3 represents the assessment of the severity of periodontal disease based on the scoring of pocket depth and comparison among the groups.

## Discussion

During training and conflict, soldiers frequently find themselves in situations where they are deprived of normal living and working circumstances, leading to stress which results in poor diet, poor hygiene, and stress [21]. They are kept in battlefield conditions, which simulate real, military operations in controlled hostile conditions, in order to ensure combat readiness. Hence, they are at high risk of development of various health problems including oral health problems owing to the nature of their work which demands utmost psychological and physical involvement. These acute or chronic oral problems may have an impact on the combat readiness which is of significant concern to them. Although there is a wealth of literature on the topic of stress in the workplace, there is surprisingly little study on how stress affects the periodontal health of Indian Army troops. Thus, the current research aimed to assess the relationship between occupational stress and periodontal health among army troops in the Indian city of Secunderabad. It can be seen from the results that the majority of them were under moderate to high occupational stress and also suffering from periodontal health problems.

The mean age of the study participants was 28.4 years which is almost similar to a study conducted on armed forces personnel (30.4 years) in Bengaluru city [6]. More than twice as many participants in the current research experienced bleeding gums than had been documented in previous studies evaluating the oral health of military members (12.3% vs 1.25%, respectively) [6,8] probably due to poor oral hygiene maintenance. Another study conducted on Indian army personnel showed that 56% had a combination of healthy gums plus bleeding-on-probing as their worst CPI score [22]. In the present study, while assessing the severity of periodontal disease (Figures 2, 3) on a scale of 0 to 2, Group A personnel had a maximum number of 0 scores (absence of condition or pocket)/Stage 1 Grade A followed by Groups D, B, and C (Stage I Grade A). Group B personnel had a maximum number of score 1 (pocket of 4-5mm)/Stage II Grade B followed by Group C (Stage II Grade A), D (Stage II Grade A), and A (Stage II Grade B). Group C personnel had a maximum number of score 2 (pocket depth of 6mm or more)/Stage III Grade A followed by A (Stage III Grade B), D (Stage III Grade B), and B (Stage III Grade B). Overall maximum number of subjects in each group has a score of 0 followed by score 1. Score 2 was seen in only a few subjects with an absence in Group B. On carrying out analysis of intergroup comparison on CLOA in teeth numbers 16, 11, 26, 46, 31, and 36 between Groups A and C (Table 2) it is revealed that more number of recruits have a score of 0 (CAL = 0-3) as compared to Group C (serving army personnel). Group C has more number of scores of 2 (CAL = 6-8mm) and 1 (CAL = 4-5mm) as compared to recruits. When a comparison is made between Groups B and D it is revealed that the majority of trainee officers/cadets have a score of 0 as compared to serving officers who are in Group D. Group D officers have more score of 1 and 2 as compared to Trainee Officers/Cadets of Group B. Further, if the analysis is made based on age and service seniority wise, then it is revealed that both Groups A and B which comprise young recruits and trainee officers/cadets have more 0 score (CAL = 0-3mm) as compared to Groups C and D which comprise serving army personnel and serving officers respectively. Overall, periodontal diseases were more prevalent in Group C followed by Groups D, B, and A. This could possibly be due to the cumulative effect of stressors over the years and the possible influence of age.

Previously conducted studies on army personnel in India have shown a lesser prevalence of shallow and deep pockets among Indian army personnel (1.3-10% for shallow pockets and 0-3% for deep pockets respectively) [6,8,22]. These results indicate that the periodontal status of army personnel in the present study was compromised. It is important to highlight that poor oral hygiene and the nature of the task may function as triggers for acute and painful periodontal disorders in a combat environment, and that daily attention to meticulous oral hygiene is required for the prevention and treatment of periodontal disease [9,10].

By safeguarding India and its people from foreign aggression and domestic instability, the Indian army takes on a tremendous duty and faces a wide variety of obstacles. These situations often lead to stressful conditions, especially psychological stress. There is a dearth of literature assessing occupational stress among Indian Army personnel. More than half of the present study population (57.7%) were under high stress. A similar finding emerged from research on the stress levels of Indian military personnel. According to the results, many service members feel that they are working too hard, are unhappy with their workplace, find their jobs dull at times, and spend too much time on tasks that are not required [22]. Another study investigated the causes of occupational stress in the Indian military, and its authors concluded that a lack of control in the workplace, excessive workload and job pressure, role conflict, inadequate awareness of one's profession, insufficient sleep, isolation, inability to express oneself, a dearth of emotional support, unrealistic goals, insufficient rest breaks, and high levels of stress hormones were the most significant contributors [19,22-24].

Research has linked psychological and social stress to an increased likelihood of developing periodontal disease [24-28]. The results of the study indicate that a considerable amount of study participants were under moderate to high occupational stress and had moderate levels of periodontal diseases. The risk of developing periodontal disease increases with an increase in occupational stress levels. Increased stress leads to reduced salivary secretion and increased plaque formation [29]. Most of the army personnel stay away for months together from their home and family members which may result in emotional stress. Saliva's pH and chemical composition/immune modulating impact, such as IgA production, are altered in response to emotional stress. As discussed earlier, stress influences the periodontium either directly or indirectly [21]. The results of the study could not be compared with any other study since no previous study was found that tried to evaluate the association between occupational stress and periodontal condition among army personnel. However, a literature search revealed other studies tested the association of stress and periodontitis among other professional groups. Japanese employees at a crane manufacturing firm were surveyed for research on the links between job stress, coping strategies, and periodontitis. An elevated periodontitis risk was seen in those who reported high levels of stress and inadequate coping mechanisms (OR = 2.79, 95% CI = 1.05-7.43, p = 0.039) [30]. Researchers in India found that employment insecurity, economic stress, a high plaque index, and other risk factors for periodontitis all go hand in hand [26].

Indian Army service should strengthen its existing oral health care services by focusing more on preventive services to improve the oral health of army personnel by evaluating their dietary pattern, oral hygiene practices, lifestyle factors, stress levels, and other factors. Furthermore, stress-reducing measures such as support from seniors and organizations and improving the social life of army personnel are necessary.

Limitations

The present study did not assess other factors like oral hygiene practice, bruxism, and salivary cortisol levels for stress evaluation. The female population was not included in the study. The study was carried out in and around Secunderabad to use its conclusive results as a further direction for large-scale nationwide study.

## Conclusions

It can be concluded that many army personnel under study were suffering from gingival bleeding and periodontal diseases. They were under considerable occupational stress. Participants under moderate to high stress were at more risk of developing periodontal diseases as compared to participants under low stress. In spite of the stress, because of the military discipline and oral hygiene motivation provided in the Indian army, their periodontal status was better in comparison to the results seen in the civilian population. Mandatory annual medical and dental checkups, regular dental camps and availability of military dental centers, and easy accessibility to dental care can be the reasons for the results obtained in this study. A weak association was seen between stress levels and periodontal status.

## Appendices

Stress Index Questionnaire

Instructions

The questionnaire consists of some statements that employees say or feel about various components and conditions of their job. You are required to select any one of the following ‘five responses’ to indicate the extent to which you agree or disagree with each statement to describe the nature and conditions of your job and also your own experiences and feelings about your job [17,18].

Q1. I have to do a lot of work in this job.

Strongly Disagree

Disagree

Undecided

Agree

Strongly Agree

Q 2. The available information relating to my job-role and its outcomes are vague and insufficient.

Strongly Disagree

Disagree

Undecided

Agree

Strongly Agree

Q3. My different officers often give contradictory instructions regarding my work.

Strongly Disagree

Disagree

Undecided

Agree

Strongly Agree

Q4. Sometimes it becomes complied problem for me to make adjustment between political / group pressure and formal rules and regulations

Strongly Disagree

Disagree

Undecided

Agree

Strongly Agree

Q5. The responsibility for the efficiency and productivity of many employees is thrust upon me

Strongly Disagree

Disagree

Undecided

Agree

Strongly Agree

Q6. Most of my suggestions are followed and implemented here.

Strongly Disagree

Disagree

Undecided

Agree

Strongly Agree

Q7. My decisions regarding distribution of assignments among employees are properly followed.

Strongly Disagree

Disagree

Undecided

Agree

Strongly Agree

Q8. I have to work with persons whom I like.

Strongly Disagree

Disagree

Undecided

Agree

Strongly Agree

Q9. My assignments are of my nature.

Strongly Disagree

Disagree

Undecided

Agree

Strongly Agree

Q10. Higher authorities do care for my self-respect.

Strongly Disagree

Disagree

Undecided

Agree

Strongly Agree

Q11. I get less salary in comparison of the quantum of my labour / work

Strongly Disagree

Disagree

Undecided

Agree

Strongly Agree

Q12. I do my work under tense circumstances

Strongly Disagree

Disagree

Undecided

Agree

Strongly Agree

Q13. Owing to excessive load I have to manage with insufficient number of employees and resources

Strongly Disagree

Disagree

Undecided

Agree

Strongly Agree

Q14. The objectives of my work role are quite clear and adequately planned.

Strongly Disagree

Disagree

Undecided

Agree

Strongly Agree

Q15. Officials do not interfere with my jurisdiction and working methods

Strongly Disagree

Disagree

Undecided

Agree

Strongly Agree

Q16. I have to do some work unwillingly owing to some group / political pressures.

Strongly Disagree

Disagree

Undecided

Agree

Strongly Agree

Q17. I am responsible for the future of a number of employees

Strongly Disagree

Disagree

Undecided

Agree

Strongly Agree

Q18. My co-operation is frequently sought in solving administrative or industrial problems at higher level.

Strongly Disagree

Disagree

Undecided

Agree

Strongly Agree

Q19. My suggestions regarding training programmes of employees are given due significance.

Strongly Disagree

Disagree

Undecided

Agree

Strongly Agree

Q20. Some of my colleagues & juniors try to defame and malign me as unsuccessful

Strongly Disagree

Disagree

Undecided

Agree

Strongly Agree

Q21. I get ample opportunity to utilize my abilities & experience independently

Strongly Disagree

Disagree

Undecided

Agree

Strongly Agree

Q22. This job has enhanced my social status

Strongly Disagree

Disagree

Undecided

Agree

Strongly Agree

Q23. I am seldom rewarded for my hardwork& efficient performance.

Strongly Disagree

Disagree

Undecided

Agree

Strongly Agree

Q24. Some of my assignments are quite risky & complicated

Strongly Disagree

Disagree

Undecided

Agree

Strongly Agree

Q25. I have to dispose off my work hurriedly owing to excessive workload.

Strongly Disagree

Disagree

Undecided

Agree

Strongly Agree

Q26. I am unable to perform my duties smoothly owing to uncertainty & ambiguity of the scope of my jurisdiction.

Strongly Disagree

Disagree

Undecided

Agree

Strongly Agree

Q27. I am not provided with clear instructions & sufficient facilities regarding the new assignments entrusted to me.

Strongly Disagree

Disagree

Undecided

Agree

Strongly Agree

Q28. In order to maintain group conformity sometimes I have to do more than the usual.

Strongly Disagree

Disagree

Undecided

Agree

Strongly Agree

Q29. I bear the great responsibility for the progress & prosperity of this organization

Strongly Disagree

Disagree

Undecided

Agree

Strongly Agree

Q30. My opinions are sought in framing important policies of the organization / department.

Strongly Disagree

Disagree

Undecided

Agree

Strongly Agree

Q31. Our interest and opinion are duly considered in making appointments for important posts.

Strongly Disagree

Disagree

Undecided

Agree

Strongly Agree

Q32. My colleagues do cooperate me voluntarily in solving administrative and industrial problems.

Strongly Disagree

Disagree

Undecided

Agree

Strongly Agree

Q33. I get ample opportunity to utilize my aptitude and proficiency properly.

Strongly Disagree

Disagree

Undecided

Agree

Strongly Agree

Q34. My higher authorities don’t give due significance to my post and work.

Strongly Disagree

Disagree

Undecided

Agree

Strongly Agree

Q35. I often feel that this job has made my life cumbersome.

Strongly Disagree

Disagree

Undecided

Agree

Strongly Agree

Q36. Being too busy with official work. I am not able to devote sufficient time to my domestic & personal problems

Strongly Disagree

Disagree

Undecided

Agree

Strongly Agree

Q37. It is not clear what type of work & behavior my higher authorities & colleagues expect from me.

Strongly Disagree

Disagree

Undecided

Agree

Strongly Agree

Q38. Employees attach due importance to formal working procedures

Strongly Disagree

Disagree

Undecided

Agree

Strongly Agree

Q39. I am compelled to violate formal and administrative procedures and policies owing to group / political pressures.

Strongly Disagree

Disagree

Undecided

Agree

Strongly Agree

Q40. My opinion is sought in changing or modifying the working system, instrument and conditions.

Strongly Disagree

Disagree

Undecided

Agree

Strongly Agree

Q41. There exists sufficient mutual cooperation & team spirit among the employees of this organization / department

Strongly Disagree

Disagree

Undecided

Agree

Strongly Agree

Q42. My suggestions and cooperation are not sought in solving even those problems for which I am quite competent.

Strongly Disagree

Disagree

Undecided

Agree

Strongly Agree

Q43. Working conditions are satisfactory here from the point of view of our welfare and convenience.

Strongly Disagree

Disagree

Undecided

Agree

Strongly Agree

Q44. I have to do such work as ought to be done by others.

Strongly Disagree

Disagree

Undecided

Agree

Strongly Agree

Q45. It becomes difficult to implement all of a sudden, the new dealing procedures and policies in place of those already in practice.

Strongly Disagree

Disagree

Undecided

Agree

Strongly Agree

Q46. I am unable to carry out my assignment to my satisfaction on account of excessive load of work and lack of time.

Strongly Disagree

Disagree

Undecided

Agree

Strongly Agree

Q47. Inability to talk openly

Strongly Disagree

Disagree

Undecided

Agree

Strongly Agree

Q48. Unhappy working relationships

Strongly Disagree 

Disagree

Undecided

Agree

Strongly agree

Q49. Lack of mutual trust and supportive culture

Strongly Disagree

Disagree

Undecided

Agree

Strongly Agree

Q50. Inadequate training for continuous career development

Strongly Disagree

Disagree

undecided

Agree

Strongly agree

Q51. Lack of identification and incorporation of new training needs program

Strongly Disagree

Disagree

Undecided

Agree

Strongly Agree

Q52. Inadequate rest breaks

Strongly Disagree

Disagree

Undecided

Agree

Strongly Agree

Q53. There is too much of competition in army

Strongly Disagree

Disagree

Undecided

Agree

Strongly Agree

Q54. Being away from my family impairs, my working ability and thinking

Strongly Disagree

Disagree

Undecided

Agree

Strongly Agree

Q55. Facing counter insurgency increases my mental strain and anxiety

Strongly Disagree

Disagree

Undecided

Agree

Strongly Agree

Q56. Deployment causes increase in my mental strain and anxiety

Strongly Disagree

Disagree

Undecided

Agree

Strongly Agree
